# Assessing Species Distribution Using Google Street View: A Pilot Study with the Pine Processionary Moth

**DOI:** 10.1371/journal.pone.0074918

**Published:** 2013-10-09

**Authors:** Jérôme Rousselet, Charles-Edouard Imbert, Anissa Dekri, Jacques Garcia, Francis Goussard, Bruno Vincent, Olivier Denux, Christelle Robinet, Franck Dorkeld, Alain Roques, Jean-Pierre Rossi

**Affiliations:** 1 Unité de Recherche 633 Zoologie Forestière, Institut National Recherche Agronomique, Centre d'Orléans, France; 2 Unité Mixte de Recherche 1062 Centre de Biologie pour la Gestion des Populations, Institut National Recherche Agronomique, Centre de Montpellier, France; Umea University, Sweden

## Abstract

Mapping species spatial distribution using spatial inference and prediction requires a lot of data. Occurrence data are generally not easily available from the literature and are very time-consuming to collect in the field. For that reason, we designed a survey to explore to which extent large-scale databases such as Google maps and Google street view could be used to derive valid occurrence data. We worked with the Pine Processionary Moth (PPM) *Thaumetopoea pityocampa* because the larvae of that moth build silk nests that are easily visible. The presence of the species at one location can therefore be inferred from visual records derived from the panoramic views available from Google street view. We designed a standardized procedure allowing evaluating the presence of the PPM on a sampling grid covering the landscape under study. The outputs were compared to field data. We investigated two landscapes using grids of different extent and mesh size. Data derived from Google street view were highly similar to field data in the large-scale analysis based on a square grid with a mesh of 16 km (96% of matching records). Using a 2 km mesh size led to a strong divergence between field and Google-derived data (46% of matching records). We conclude that Google database might provide useful occurrence data for mapping the distribution of species which presence can be visually evaluated such as the PPM. However, the accuracy of the output strongly depends on the spatial scales considered and on the sampling grid used. Other factors such as the coverage of Google street view network with regards to sampling grid size and the spatial distribution of host trees with regards to road network may also be determinant.

## Introduction

Globalisation and one of its most publicized consequences, the species redistribution, has received a considerable attention during the last decades [1]–[3]. A lot of research efforts has been directed towards unravelling ecological processes implied in the spread of species [2], [4]. In that context, GISs have proven particularly precious to monitor species spread [5]. There is a concomitant increasing demand for data documenting the spatial distribution of species for different objectives such as monitoring and modelling species range expansion [6], [7], anticipating future distributions and devising control strategies [8]–[11], studying mechanisms at work with species dispersal and the relationships with landscape composition and physiognomy [12]. Unfortunately, the amount of data available is limited for a majority of taxa and geographical regions. The consequences of data scarcity are dramatic for example in the field of species distribution modelling [13], [14] where collecting occurrence data is not always easy, time consuming, and is often non-environmentally friendly (because of the gas emission of vehicles used for the survey). As a result, updating or completing existing data sets is difficult albeit it is the very first step of ecological analysis and modelling.

During the last decade, geospatial data have become increasingly accessible with the advent of new mapping technologies such as Google Earth that offers free satellite imagery and aerial photos of most of earth's land surface. Google Earth has been used in several research areas that require mapping technology such as human or animal health [15], [16], conservation biology [17], [18] or biodiversity assessment [19]. A new level of spatial information has been recently reached with the development of Google street view (GSV) in 2007 [20]. This new technology provides panoramic imagery captured in hundreds of cities in different countries around the world. It corresponds to an unprecedented amount of information at street-level scale. Not only dedicated to cities and urban areas, GSV documents rural areas and unpopulated places. GSV is based on the idea of operating numerous data-collection vehicles around the world. Each vehicle is equipped with camera and GPS, and records images while driving paved and unpaved roads. Resulting data are processed and served via the Internet [20]. Street imagery consists of detailed views allowing users to navigating and exploring streets and cities [21]. The aim of the present study was to explore how the GSV technology could be helpful to ecological research in documenting the geographical distribution of species. Recent studies have shown that the GSV imagery could be used to depict and audit neighborhood environments in the framework of social science [22] and preventive medicine [23] but to our knowledge, no ecological application has been published so far.

We assessed the presence of an insect species by roadside sampling [24] based on GSV imagery and compared the outputs with independent field data. We selected the Pine Processionary Moth (PPM) (*Thaumetopoea pityocampa* Den. & Schiff., Lepidoptera, Notodontidae) as the biological model for our survey because it is a good example of expanding species that offers various advantages with regard to our aims. The PPM is a pine defoliator occurring on various tree species of the genera *Pinus* and *Cedrus* that are used in forestry or as ornamental trees in urban and rural areas throughout Europe. PPM larvae build white winter nests that are easily discernable and thus provide unambiguous indication of species presence since no other organism produce similar structures in these tree species at that time of the year. The nests are spatially aggregated [25] and exhibit a strong edge effect with considerably higher densities at stand edges [26] or along host tree-lined streets. This characteristic is invaluable with regards to roadside sampling. We sampled the subject species in two sampling areas using both field sampling and visual examination of GSV imagery and worked at two resolutions (*i.e*. grains) in order to test possible scale effects. Attention was paid to the coverage of the GSV database because it could constitute an important cause of discrepancies between field and *in silico* data sets. This study is a first step towards new methodologies for monitoring species geographical distribution across large spatial scales making use of the ever-increasing amount of data available through the Internet.

## Materials and Methods

### Model species

The PPM is a common defoliator occurring on various native and exotic conifer species throughout southern Europe and Mediterranean countries, where it is the most important pine and cedar defoliator. Its preferred host, *Pinus nigra*, as well as several other potential host tree species have been used for both large-scale afforestation and ornamental plantations and are thus widely distributed. The PPM range is largely controlled by the minimum winter temperatures [27], [28]. A recent study has revealed that the PPM geographical range is expanding both northward and in altitude [29], probably in relation to climate changes [11], [27], [30], [31]. Adult emergence occurs during summer depending on local climatic conditions. Soon after emergence, adults mate and females select a host tree and lay one egg batch on the host tree needles. Hatching occurs roughly one month later *i.e*. from August to September in our study area. Larvae are gregarious, feed on pine needles, and build a silk nest [32], [33]. The first two instars build small temporary silk nests only detectable from nearby the host tree. From the third instar on, larvae built a definitive nest in which they will develop during autumn and winter. The winter nest is white and shiny due to newly produced silk. The pupation procession (which gave its name to the species) is the migration of larvae into the soil where they pupate until the following summer. It occurs at the end of winter or in early spring according to climatic and meteorological conditions. Empty nests turn to brownish and deteriorate. They have usually disappeared the following year when next PPM generation starts to build new nests.

### Ethics statements

All the data used in the present work were collected on the public thoroughfare and thus did not require specific permissions according to the French law (Arrêt n. 516 du 7 mai 2004 Cour de cassation – Assemblée plénière). The present survey did not involve endangered or protected species.

### Sampling zones and grid resolution

**Figure 1:**
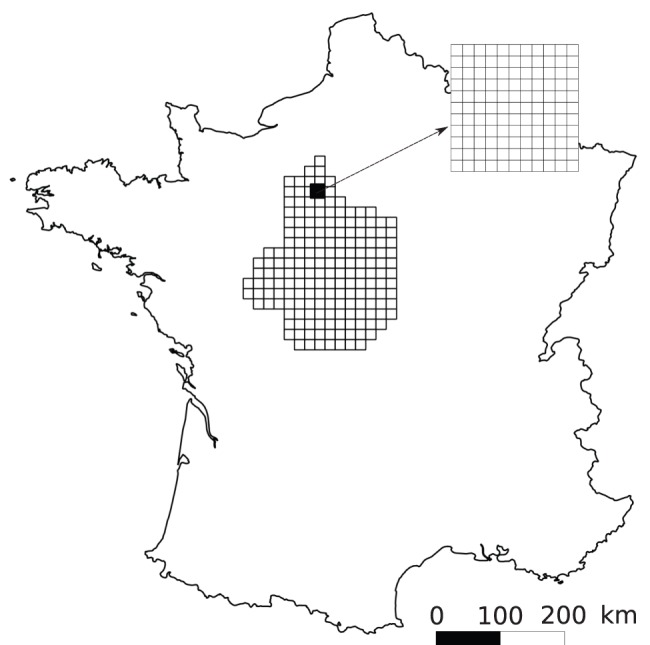
Pine processionary moth sampling grids. A large sampling grid covering the administrative region called “Région Centre” (46 848 km^2^) in France was investigated. A second, smaller (121 km^2^), sampling grid was nested within the former.

We surveyed the PPM spatial distribution in two sampling areas of contrasted extent (Figure 1). A large sampling area (46 848 km^2^) was designed so as to cover the Région Centre in France which northern and eastern parts have been recently or are currently being colonized by the PPM. A second, smaller sampling area (22×22 km  = 484 km^2^) was located within the former at the north of the Beauce area. Each sampling area was discretized into a set of sampling cells which size defined the grain or resolution of the survey. The large sampling area was discretized into 183 cells of 16×16 km size (Figure 1). This sampling zone will be thereafter referred to as LG (large grid). Similarly, the Beauce sampling window was discretized into a set of 121 cells of 2×2 km size and the resulting sampling grid will be referred to as SG (small grid) (Figure 1).

### Field sampling protocols

The PPM was sampled within each individual cell throughout each sampling grid according to a protocol defined to monitor the species range expansion towards northern France [30], [34] on the basis of nest road sampling. Each cell is visited by car and PPM host trees are observed by eye and with binoculars (when necessary) from the road and public land. When a nest is observed, it is georeferenced, the species is considered as present in the cell and then the neighbouring cells are prospected. When all the paved and unpaved roads practicable by car within a cell have been visited without detecting a nest, the PPM is considered as absent. The geographic coordinates of the tree hosting the observed nest are recorded by GPS (or the location of this tree is mapped onto a georeferenced aerial photo using PhotoExploreur or Arpentgis mobile in case of distant observation).

PPM presence-absence data in both grids were collected between 2007 and 2009 following the protocol described above. Note that a grid cell where the PPM was sighted on one sampling occasion is assumed to remain colonized the following years.

### Sampling based on GSV

**Figure 2:**
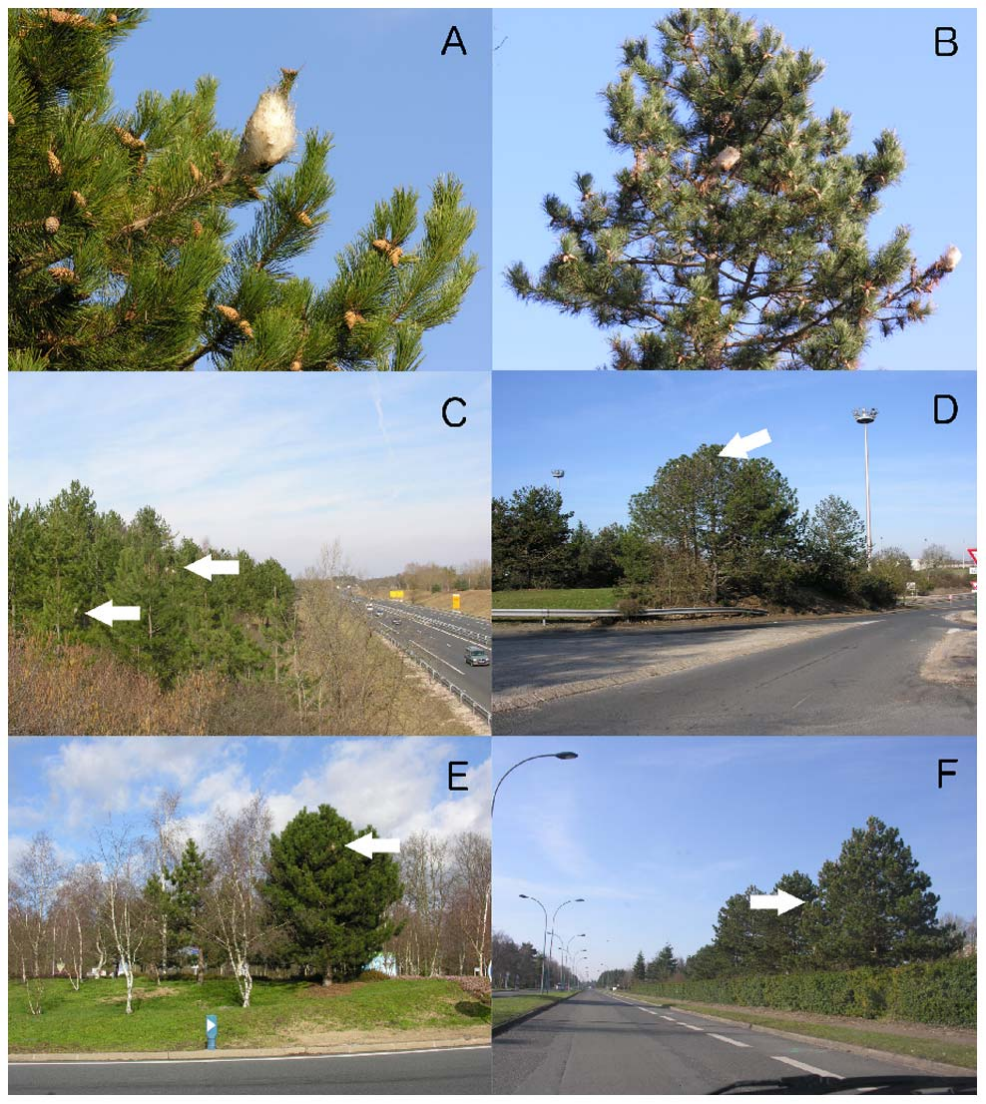
Pictures showing the pine processionary moth silk nest and different examples of infested trees located along streets in the region of Orléans, France. A. Winter silk nest. B. Host tree infested by several PPM colonies. C to E. Infested trees located along traffic lanes. F. Picture taken from within a car. All host trees are black pines (*Pinus nigra*) except in C. where black pines and scots pines (*P. sylvestris*) are present. All photos by J. Rousselet.

For each cell, an operator virtually drove along the roads available in the GSV database. He performed *in silico* roadside sampling by visually analyzing the available panoramic views. Figure 2 shows different pictures of PPM nests, infested trees and several trees located along streets in the region of Orléans, France, as they could be observed using GSV. When different views of the same place were available, all the points of view were explored. This work was realized in September and October 2011. As soon as a nest was detected, the cell was considered colonised by the PPM and the operator switched to another cell.

When a nest was difficult to identify, several views with different zoom levels and different viewing angles were examined. The spatial coordinates of each nest were recorded. Four kinds of cells were thus considered: cells without road covered by GSV at the date of the observation; cells containing at least one nest identifiable without ambiguity (“presence” cell); cells with a putative nest but with a persistent doubt (“indistinct” cells); cells without nest detected (“absence” cells).

### Spatial coverage of Google roads and GSV

For the SG, we encountered several cells with no available GSV data and a high discrepancy between field and *in silico* data (see results). We therefore assessed the structure of the road network covered by GSV to examine its spatial variability and determine if it could explain the divergences between data sets. We developed a java script to collect information from the Google API (code available in Text S1), we then derived maps of roads included in the GSV database on a regular grid of 250 by 250 m mesh. The coordinates of each point were used to retrieve the nearest road and the nearest road available in the GSV database. We additionally computed an index to quantify the coverage by GSV within each sampling cell using the following procedure: cells were rasterized at the resolution of 250 by 250 m using the R package raster [35]. We then computed the proportion of pixels corresponding to road covered in the GSV database.

### Comparing field and Google-derived data sets

Both zones led to two grids of PPM occurrences corresponding to field and Google-derived data. This was considered as a two-class prediction problem *i.e*. binary classification. Cells in which the PPM was observed formed the “positive” class while cells where the PPM was absent formed the “negative” class. We computed the four possible outcomes of that binary classifier, namely the true positive (TP), the true negative (TN), the false positive (FP) and the false negative (FN) [36], assuming that field sampling gave true data. TP corresponded to cases where the PPM was observed both in the field and from Google database. TN corresponded to absence in the field and in the Google derived data. FP corresponded to cells where the PPM was not observed in the field but was present according to Google data. Finally FN corresponded to field observations associated to absence according to Google data. TN, FN, TP and FP formed the confusion matrix reported in the results section (Tables 1 and 2). The sensitivity (rate of TP) and the specificity (rate of TN) were used to measure the proportion of good predictions derived from Google data in the case of cells where PPM is present and absent respectively. The sensitivity and the specificity were estimated as TP/P and TN/N where P and N are the total number of positive and negative cases respectively. We computed the accuracy or the rate of good predictions as (TP+TN)/(P+N). We additionally computed the Phi/Matthews correlation coefficient as a measure of discrete covariation between field and Google derived data [37].

**Table 1:** Confusion matrix for the pine processionnary moth field data (true class) and Google-derived data (hypothetized class) in the large-scale study grid (LG).

		field data	
		presence	absence
Google	presence	TP = 165	FP = 0
	absence	FN = 13	TN = 5

**Table 2:** Confusion matrix for the pine processionnary moth field data (true class) and Google-derived data (hypothetized class) in the small-scale study grid (SG).

		field data	
		presence	absence
Google	presence	TP = 3	FP = 0
	absence	FN = 63	TN = 49

Of the 121 sampled cells, 6 were removed from the analysis because no GSV data were available for comparison with field data.

This coefficient ranges from 1 to +1 with 1 indicating a perfect prediction, 0 a random prediction and negative values a worse than random prediction. All computations were done using the R statistical software [38] and the R package ROCR [37].

## Results

### Large sampling grid

**Figure 3:**
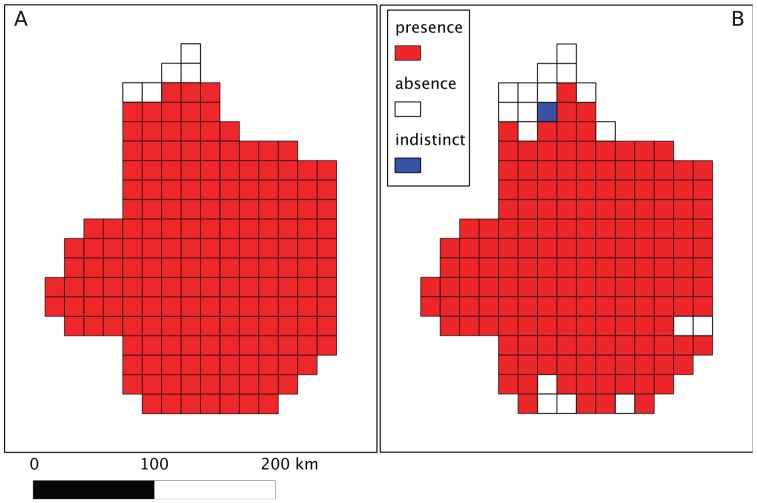
Large-scale study of the pine processionnary moth in France. A. Field data B. Google street view derived data. The sampling units are cells of 16×16 km.

The field data collected for the large scale survey showed that the PPM was present all over the study area: there were 178 cells classified as “presenc” and 5 as “absence”. Google-derived data provided slightly different values with 165 cells classified as “presence” and 17 as “absence”. One cell was reported as “indistinct” because the pictures did not allow ascertaining the presence of PPM nests (Figure 3). In that case, we assigned the status of absence to the cell when computing sensitivity and other indices. Off the 183 cells, 165 were true positives and 5 were true negatives (Table 1). The number of false negatives was 13 and there was no case of false positive (Table 1). This led to very high values of the sensitivity (true positive rate) and specificity (true negative rate), which were respectively 0.927 and 1. Both field and Google-derived data are reported in Figure 3. Overall, Google-derived data showed a good agreement with field data as revealed by an accuracy of 0.929 and a Matthews correlation coefficient of 0.507.

### Small sampling grid

**Figure 4:**
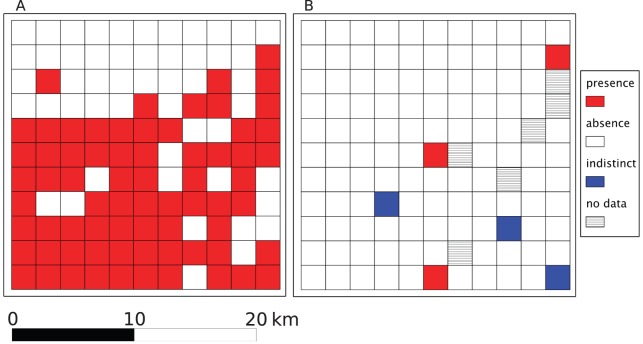
Small-scale study of the pine processionary moth in France. A. Field data B. Google street view derived data. The sampling units are cells of 2×2 km.

The field data collected over the SG consisted in 70 and 51 cells classified as “presence” and “absence” respectively. Google-derived data comprised 3 cells classified as “presence”, 109 cells where the PPM was absent, 3 indistinct cells, and 6 cells for which no GSV data were available (Figure 4). As in the case of the large-scale survey, the “indistinct” cells were classified as “absence” and we did not account for the cells with no data in the computation of the statistics.

There were 6 true positives and 49 true negatives while the number of false negatives was 63 and there was no case of false positive (Table 2). As a consequence, these values led to a low sensitivity of 0.045 while the specificity was 1. The Google-derived data showed a fairly low agreement with field data as the accuracy was 0.452 and the Matthews correlation coefficient was 0.141.

### Linking GSV coverage and the PPM sampling

**Figure 5:**
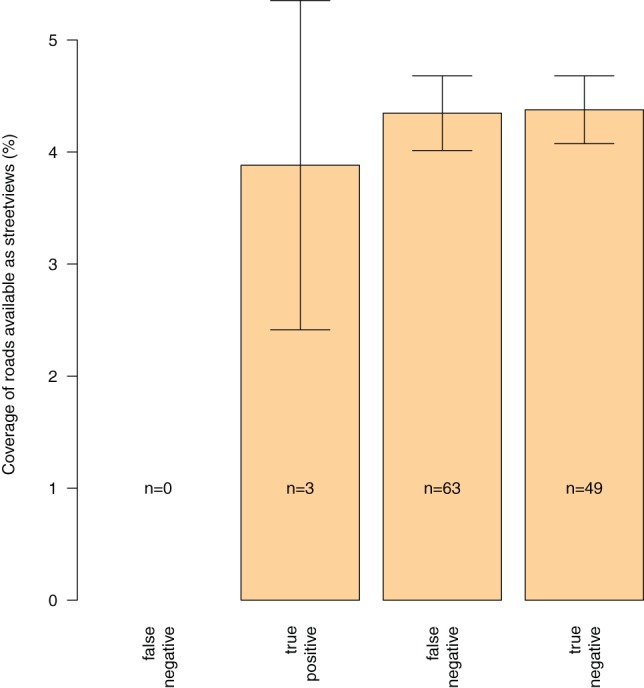
Relationships between the Google street view coverage (%) and the counts of true and false positives, and true and false negatives in the SG.

The coverage of the network was expressed as the proportion of pixels corresponding to roads within each sampling cell. Overall, this network covered the whole sampling area, with higher coverage around the main towns like in the bottom right part of the map that corresponds to the surroundings of the city of Chartres. The importance of the GSV coverage was not related to the number of true positive, false negative or true negative cases as shown in Figure 5. Note that the percentage used here is not the proportion of roads available within GSV but the amount of pixels (250 by 250 m) corresponding to roads available within GSV.

## Discussion

### Spatial scale issues: resolution

The present study showed that the data derived from GSV imagery were good surrogates for field data when assessing the spatial distribution of the PPM. By comparing Google-derived and field data, however, we showed that the resolution of the survey was critical in that fine scale *i.e*. high resolution sampling failed to properly describe the actual distribution (*viz* as perceived with field sampling). Scale effects are well known in ecology [39] and in our case they are directly linked to the resolution or grain, *i.e*. to the mesh of the sampling grid (Figure 1). It is analogous to the size of the sampling unit, which has been shown to strongly affect our perception of spatial variability [40], [41] both in intensity and range.

Google-derived data depend on the density of the GSV coverage which parameter in turn depends on the size of the sampling units. Large sampling units such as squares of 16×16 km used in the LG survey are more likely to include a large amount of roads covered in the GSV system which implies more chance to properly spot PPM nests when present. Decreasing the grain amounts to increasing the sampling effect with lower level of information per unit and ultimately no information in some locations where no street views are available (we reported 6 such cases in the SG). Such effect may become less meaningful in the future, as the density of the Google coverage will be progressively enhanced.

The grain also affects the variability of the date of the Street views picture, which may be a central parameter for some species as the PPM (see below).

### Date of pictures: seasonality and other sources of uncertainty

Some organisms may concentrate or be visible at particular season or times of the day and this defines the periods when census becomes possible. Because we used the Google views (pictures) as a data source, we depend on the date at which pictures where taken. This is of a particular importance in the case of the PPM because nests can mostly be observed during winter and deteriorates after larvae migration into the soil. More generally, using GSV for species assessment should be approached with caution when species census is seasonal. As evoked above, the uncertainty introduced by processing pictures taken at various dates along the year is increasing as the grain is decreasing. It is important to highlight the value of the Google data for future research. Google database is updated and this growing body of data will constitute a tremendous amount of information in the next decades allowing for example researchers to perform retrospective analyses of species expansion processes and biological invasions. Chen et al. [42] recently published the results of a study assessing 100 years of environmental changes in Western China based on the comparison of modern versus 100-years old pictures of 250 localities from Western China. They showed how this type of data could help detecting and characterising changes in vegetation, landscape and more globally biodiversity. We believe that GSV may contribute to such type of retrospective surveys with unprecedented power and resolution.

### Potential of GSV regarding species monitoring

As underlined above, only species visible from photographs or associated with conspicuous sign of presence can be surveyed using GSV imagery. This encompasses various organisms that alter significantly and specifically the colour or shape of trees as well as tree and plant species that colonize road edges or can be reliably sighted from roads. With that regards, the PPM constitutes a good biological model since its winter nests are white and easily visible in the tree foliage. In addition, PPM spatial aggregation at the edges of forest plots or along roads [26] increases the probability that the species be spotted if present in a given area. The present study focused on presence/absence data and the potential use of GSV for density census has not been considered. This question is more complicated and requires an estimate of the prospected surface [43]. Although GSV initially focused on city streets it was quickly extended to peri-urban areas and is now increasingly available for rural areas including large agricultural regions with low population densities. This is the case for different parts of our study region and in particular the Beauce area (Figure 4). The results reported in the present paper show that the GSV system, in its present form, provides a visual overview of urban, peri-urban and rural streets network allowing a proper assessment of the PPM regional distribution when assessed using large sampling window (here, 16×16 km). It is likely that the value of the GSV system will increase in the future as the density of the network will increase and that, for that reason, studies based on finer resolution will become possible.

In fact, the better representativeness of urban areas in GSV may constitute an opportune bias. Populated places often constitute major points of establishment for exotic organisms [44] from which invasive species might disperse towards other areas using different types of corridors [45]. Species monitoring in urban areas using GSV may prove useful to identify spots of invasive species, their dispersal pathways and the potential landscape features that slow down or speed up their dispersal.

### Conclusions

The present survey illustrated how the GSV imagery could be used to perform *in silico* sampling of species occurrences. It must be emphasized that only organisms that can be reliably detected by road sampling can be assessed using GSV. The case of the PPM is straightforward as this species produces easily visible winter nests but many other organisms might require important calibration efforts. The scale issue deserves to be considered carefully and it must be noted that the method might perform poorly at small grains (high resolution). Although the ever-increasing coverage of GSV system should improve the method performances, we have no clue as to when and where small-scale sampling would become accurate. With only one case study, we obviously lack hindsight to advertise the technique for general use but our results show that it has some promise for future use, at least with species easily observed by means of road sampling such as the PPM.

## Supporting Information

Text S1 **The java script code used to identify the roads covered in the Google street view database.**
(TXT)

## References

[1] Lin W, Zhou G, Cheng X, Xu R (2007) Fast economic development accelerates biological invasions in China. PLoS ONE 2: e1208. 18030342 10.1371/journal.pone.0001208 PMC2065902

[2] Lockwood JL, Hoopes MF, Marchetti MP, editors (2007). Invasion ecology. Oxford: Blackwell Publishing. 304 p.

[3] Perrings C, Mooney H, Williamson M, editors. (2010) Bioinvasions and globalization. Ecology, economics, management, and policy. Oxford: Oxford University Press. 267 p.

[4] Thomas CD, Ohlemüller R (2010) Climate change and species distributions: An alien future? In: Perrings C, Mooney H, Williamson M, editors. Bioinvasions and globalization. Ecology, economics, management, and policy. Oxford: Oxford University Press. 19–29.

[5] Clay SA, editor. (2011) GIS applications in agriculture. Volume Three: Invasive species. Boca Raton: CRC Press. 418 p.

[6] Brooker RW, Travis JMJ, Clark EJ, Dytham C (2007) Modelling species' range shifts in a changing climate: The impacts of biotic interactions, dispersal distance and the rate of climate change. J Theor Biol 245: 59–65. 17087974 10.1016/j.jtbi.2006.09.033

[7] Rey O, Estoup A, Vonshak M, Loiseau A, Blanchet S, et al (2012) Where do adaptive shifts occur during invasion? A multidisciplinary approach to unravelling cold adaptation in a tropical ant species invading the Mediterranean area. Ecol Lett 15: 1266–1275. 22906215 10.1111/j.1461-0248.2012.01849.x

[8] Franklin J (2009) Mapping species distributions: spatial inference and prediction. Cambridge: Cambridge University Press. 320 p.

[9] Horn A, Kerdelhué C, Lieutier F, Rossi J-P (2012) Predicting the distribution of the two bark beetles *Tomicus destruens* and *Tomicus piniperda* in Europe and the Mediterranean region. Agric Forest Entomol 14: 358366.

[10] Mulñoz A-R, Real R (2006) Assessing the potential range expansion of the exotic monk parakeet in Spain. Divers Distrib 12: 656–665.

[11] Robinet C, Baier P, Pennerstorfer J, Schopf A, Roques A (2007) Modelling the effects of climate change on the potential feeding activity of *Thaumetopoea pityocampa* (Den. & Schiff.) (Lep., Notodontidae) in France. Global Ecol Biogeogr 16: 460–471.

[12] Hill JK, Collingham YC, Thomas CD, Blakeley DS, Fox R, et al (2001) Impacts of landscape structure on butterfly range expansion. Ecol Lett 4: 313–321.

[13] Hernandez PA, Graham CH, Master LL, Albert DL (2006) The effect of sample size and species characteristics on performance of different species distribution modeling methods. Ecography 29: 773–785.

[14] Wisz MS, Hijmans RJ, Li J, Peterson AT, Graham CH, et al (2008) Effects of sample size on the performance of species distribution models. Divers Distrib 14: 763–773.

[15] Carvalho LFR, de Melo CB, McManus C, Haddad JPA (2012) Use of satellite images for geographical localization of livestock holdings in Brazil. Prev Vet Med 103: 74–77. 21917345 10.1016/j.prevetmed.2011.08.006

[16] Chang AY, Parrales ME, Jimenez J, Sobieszczyk ME, Hammer SM, et al (2009) Combining Google Earth and GIS mapping technologies in a dengue surveillance system for developing countries. Int J Health Geogr 8: 49. 19627614 10.1186/1476-072X-8-49 PMC2729741

[17] Asner GP, Rudel TK, Aide TM, Defries R, Emerson R (2009) A contemporary assessment of change in humid tropical forests. Conserv Biol 23: 1386–1395. 20078639 10.1111/j.1523-1739.2009.01333.x

[18] Giri C, Ochieng E, Tieszen LL, Zhu Z, Singh A, et al (2011) Status and distribution of mangrove forests of the world using earth observation satellite data. Global Ecol Biogeogr 20: 154–159.

[19] Guralnick RP, Hill AW, Lane M (2007) Towards a collaborative, global infrastructure for biodiversity assessment. Ecol Lett 10: 663–672. 17594421 10.1111/j.1461-0248.2007.01063.x PMC2040220

[20] Anguelov D, Dulong C, Filip D, Frueh C, Lafon S, et al (2010) Google street view: Capturing the world at street level. Computer 43: 32–38.

[21] Frome A, Cheung G, Abdulkader A, Zennaro M, Bissacco A, et al.. (2009) Large-scale privacy protection in Google street view. In: Proc. 12th IEEE Intl Conf. Computer Vision (ICCV 09), IEEE Press 2373–2380.

[22] Odgers CL, Caspi A, Bates CJ, Sampson RJ, Moffitt TE (2012) Systematic social observation of childrens neighborhoods using Google street view: a reliable and cost-effective method. J Child Psychol Psychiatry 53: 1009–1017. 22676812 10.1111/j.1469-7610.2012.02565.x PMC3537178

[23] Rundle AG, Bader MDM, Richards CA, Neckerman KM, Teitler JO (2011) Using Google street view to audit neighborhood environments. Am J Prev Med 40: 94–100. 21146773 10.1016/j.amepre.2010.09.034 PMC3031144

[24] Samalens J-C, Rossi J-P, Guyon D, Van Halder I, Menassieu P, et al (2007) Adaptive roadside sampling for bark beetle damage assessment. Forest Ecol Manage 253: 177–187.

[25] Arnaldo PS, Torres LM (2005) Spatial distribution and sampling of *Thaumetopoea pityocampa* (Den. & Schiff.) (Lep. Thaumetopoeidea) populations on *Pinus pinaster* Ait. in Montesinho, N. Portugal. Forest Ecol Manage 210: 1–7.

[26] Samalens J-C, Rossi J-P (2011) Does landscape composition alter the spatiotemporal distribution of the pine processionary moth in a pine plantation forest? Popul Ecol 53: 287–296.

[27] Battisti A, Stastny M, Netherer S, Robinet C, Schopf A, et al (2005) Expansion of geographic range in the pine processionary moth caused by increased winter temperatures. Ecol Applic 15: 2084–2096.

[28] Huchon H, Démolin G (1970) La bioécologie de la processionnaire du pin: dispersion potentielle-dispersion actuelle. Rev Forest Fr 22: 220–233.

[29] Battisti A, Stastny M, Buffo E, Larsson S (2006) A rapid altitudinal range expansion in the pine processionary moth produced by the 2003 climatic anomaly. Global Change Biol 12: 662–671.

[30] Robinet C, Rousselet J, Imbert C-E, Sauvard D, Garcia J, et al (2010) Le réchauffement climatique et le transport accidentel par l'homme responsables de l'expansion de la chenille processionnaire du pin. Forêt Wallonne 108: 19–27.

[31] Rosenzweig C, Casassa G, Karoly D, Imeson A, Liu C, et al.. (2007) Assessment of observed changes and responses in natural and managed systems. In: Parry ML, Canziani OF, Palutikof JP, van der Linden PJ, Hanson CE, editors. Climate change 2007: Impacts, adaptation and vulnerability. Contribution of working group II to the fourth assessment report of the intergovernmental panel on climate change. Cambridge: Cambridge University Press. 79–131.

[32] Démolin G (1969) Comportement des adultes de *Thaumetopoea pityocampa* Schiff.: dispersion spatiale, importance écologique. Ann Sci Forest 26: 81–102.

[33] Geri C, Millier C, Xeuxet D (1985) Mesure des populations de processionnaire du pin (*Thaumetopoea pityocampa* Schiff – Lépidoptère Thaumetopoeidae) au Mont-Ventoux. Ann Sci Forest 42: 143–184.

[34] Roques L, Soubeyrand S, Rousselet J (2011) A statistical-reaction-diffusion approach for analyzing expansion processes. J Theor Biol 274: 43–51. 21237178 10.1016/j.jtbi.2011.01.006

[35] Hijmans RJ, van Etten J (2012) raster: Geographic data analysis and modeling. R package version 2.0-41. Available: http://CRAN.R-project.org/package=raster. Accessed: 10 Sep 2013.

[36] Fawcett T (2006) An introduction to ROC analysis. Pattern Recog Lett 27: 861874.

[37] Sing T, Sander O, Beerenwinkel N, Lengauer T (2005) ROCR: visualizing classifier performance in R. Bioinformatics. 21: 3940–3941. 10.1093/bioinformatics/bti623 16096348

[38] R Development Core Team (2013) R: A Language and Environment for Statistical Computing. Vienna, Austria: R Foundation for Statistical Computing. Available: http://CRAN.R-project.org/. Accessed: 10 Sep 2013.

[39] Levin SA (1992) The problem of pattern and scale in ecology: The Robert H. MacArthur award lecture. Ecology 73: 1943–1967.

[40] Bellehumeur C, Legendre P, Marcotte D (1997) Variance and spatial scales in a tropical rain forest: Changing the size of sampling units. Plant Ecol 130: 89–98.

[41] Rossi J-P, Nuutinen V (2004) The effect of sampling unit size on the perception of the spatial pattern of earthworm (*Lumbricus terrestris* L.) middens. Appl Soil Ecol 27: 189–196.

[42] Chen H, Yin K, Wang H, Zhong S, Wu N, et al (2011) Detecting one-hundred-year environmental changes in western China using seven-year repeat photography. PLoS ONE 6: e25008. 21966397 10.1371/journal.pone.0025008 PMC3178569

[43] Southwood TRE, Henderson PA (2000) Ecological methods. Oxford: Blackwell Science. 575 p.

[44] Smith RM, Baker RHA, Malumphy CP, Hockland S, Hammon RP, et al (2007) Recent non-native invertebrate plant pest establishments in Great Britain: origins, pathways, and trends. Agric Forest Entomol 9: 307–326.

[45] Säumel I, Kowarik I (2010) Urban rivers as dispersal corridors for primarily wind-dispersed invasive tree species. Landscape Urban Plann 94: 244–249.

